# The Impact of Performance Enhancement on Value of Care in Hospitals

**DOI:** 10.3389/fpubh.2021.740257

**Published:** 2021-12-23

**Authors:** Peter Bertke, Martin Nufer

**Affiliations:** Hirslanden Klinik St. Anna, Lucerne, Switzerland

**Keywords:** value, performance, care, hospital, quality, cost, innovation

## Abstract

Hospitals are facing big challenges: decreasing reimbursements are going alongside increasing costs and the necessity of investments. At the same time occurring, excellent quality of care, and high-patient satisfaction have to be assured. The dilemma of providing both with decreasing rather than increasing resources cannot be solved only by striving for economies of scale, but by optimizing supply chain management, or reduction of overhead. Possible effects of these measures most often are already exhausted and seldom have a positive impact on the quality of care or patient satisfaction. Management is tempted to use its best-known instruments to reduce costs, while medical staff's focus is on quality of care and often battle against management as a perceived enemy. The solution to this dilemma lies in focusing on medical core processes that are directly linked to patients' treatments and, thereby improving all the parameters of Michael Porter's value equation: costs, outcome, and patient satisfaction. This approach of performance enhancement presumes understanding, acceptance, and constructive collaboration of two usually separated worlds: The medical-scientific world involved in patient care and the financial world of management. In this article, the authors explain performance enhancement for optimized delivery of care and how the dilemma mentioned above can be solved. The authors explain how performance enhancement can be achieved in daily clinical practice, which kind of obstacles have to be overcome, which changes are necessary within a hospital, how medical staff can be motivated, and how the value of care equation can be influenced.

## Introduction

Just imagine your salary is declining by small percentages every year while your costs of living stay steady or even rise. It is obvious that this development would present a real threat to everyone's living standard and contribute to an unsatisfactory personal situation. Very few of us would agree to sustain something valuable every coming year due to decreasing financial resources.

However, a similar situation can be found in hospitals of various countries: decreasing reimbursement is occurring alongside steady or even rising costs. At the same time, excellent quality of care and high-patient satisfaction have to be assured. The dilemma of providing both with decreasing rather than increasing resources cannot be solved by using the best-known management tools: economies of scale, optimizing supply chain management, reducing overhead, further increasing the productivity of employees, or reducing spending for both the existing and new business.

So, how can this dilemma be solved, especially against the background that the economic potential concerning waste and inefficiency in the Swiss healthcare sector is estimated to be several billion Swiss Francs (1)?

## Patient Journey and Medical Core Processes

**Figure 1:**
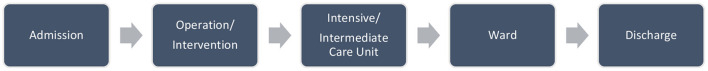
A patient journey through a hospital. Patients enter the hospital as planned or unplanned admission. They might undergo an operation/intervention, stay at an intensive or intermediate care unit if in severe medical condition, are usually treated on a ward after that, and are discharged after their medical condition has improved. At every single stage, we can look at the medical core processes and evaluate how to improve them.

The core competency and responsibility of a hospital and its medical staff are delivering care to the patients. Patients either enter a hospital as planned or unplanned admission, the latter most often *via* the emergency department (ED). They then pass through different stations on their “journey,” depending upon the kind of health issue and its severity, as seen in Figure 1.

While moving from one stage to another during their journey, patients receive a part of their diagnostic and/or therapy, planned, coordinated, and executed by the medical staff (doctors, nurses, therapists, etc.). Every single act of the medical staff for patients can be understood as a medical core process. Medical core processes, thereby, can be best described as interactions between patients and medical staff in the context of their diagnostic and/or therapeutic measures, as the following examples show:

Besides regular treatment for bacterial infection of the bladder, the evaluation of a patient's social situation at home is a medical core process as well, executed by nurses on the ED (station “Admission,” Figure 1). A precise decision about the appropriateness of costly bone tissue during a spine fusion by neurosurgeons is a medical core process as well (station “Operation/Intervention,” Figure 1). Several blood transfusions on the intensive care unit after a long and complicated abdominal surgery can also be viewed as a medical core process. Switching intravenous antibiotics to pills on the ward while treating bacterial infections of the lungs is a medical core process that involves both the doctors and nurses. During discharge, pharmaceutical support concerning discharge medication, patient education about side effects, and information on how to take pills is a medical core process as well.

Medical core processes can be found at every stage of a patient's journey, regardless of the medical discipline the patient is treated. These medical core processes should be systematically questioned, challenged, and—if possible—improved. By improvement of medical core processes as the very starting point of performance enhancement, we can improve performance on the one hand and make a positive impact on the value of care according to Michael Porter's landmark article “What is Value in Healthcare” published in 2010 (2).

## Improvement of Medical Core Processes and the Impact on the Value of Care

In this 2010 article and the previous publications, Michael Porter presents his central concept that the “value” of care a patient receives should not only be measured according to the success of a single procedure or traditional outcome measures such as mortality or rehospitalization rates. Value of care should also be assessed by taking into account so-called “outcomes that matter to patients.” These can be multidimensional and also include functional status, patient's experience, and sustainability of treatment. At the same time, care delivered to the patients should be provided at acceptable costs.

In the context of radical prostatectomy, for example, one should not only measure mortality, infection, or readmission rates but also patient-oriented outcomes such as loss of sexual function or degree of bladder function.

Value of care, therefore, is defined as the ratio of “health outcomes” on the one hand (composed of outcome measurement and patient experience) and costs on the other hand, as seen in the equation below.


$$\begin{array}{l} \ll Value\;of\;care\gg =\frac{Outcome\;+\;Patient\;Experience}{Costs} \end{array}$$

All the parameters of this equation can be influenced by a systematic approach to performance enhancement by focusing on medical core processes. In the next sections, we discuss how the improvement of medical core processes can be achieved and what impact can be made on the value of care is discussed.

### Innovation

Innovation—in short—is about problem solving. By innovation, we usually understand a new idea solving a problem or a need.

In daily clinical practice, we are confronted with various problems: hospitals are taking care of more and more elderly patients, many of whom not only have an acute medical issue but also social needs such as requiring evaluation of their domestic situation or the necessity of a post-acute care discharge solution. Most often this evaluation as a medical core process takes place later on during a stay, in the worst case shortly before discharge. However, an evaluation taking place on the day of admission could solve several problems: every hospital is dependent on timely and efficient care of patients to avoid unnecessary prolonging of the stay. Every patient needs to prepare for appropriate care after the stay as well. The so-called post-acute care discharge (PACD) score is an innovative solution that is able to solve both problems. This score is very well-established in evaluating the need for a solution after discharge within the first 24 h after admission (3). Similarly, length of stay and, therefore, costs (=denominator of the value equation above) can be reduced and the patient's experience (=numerator of the value equation) can be enhanced, thus making a positive impact on the value of care.

In the ward, many patients are treated because of infectious diseases and receive intravenous antibiotics; prescribing and administering them are both the medical core processes. However, it is well-known that the duration of intravenous antibiotic treatment for a large group of common infectious problems (e.g., a bacterial infection of the lung or bladder) often is longer than required. Switching to pills earlier if justifiable is not only less costly but also enhances the patient's experience because an intravenous line is no longer needed and mobilization is far easier without one. In addition, switching intravenous antibiotics to pills and removing intravenous lines are associated with fewer complications such as thrombophlebitis, an infection of the vein at the entry point of the intravenous line. An innovative, IT-supported solution to switch intravenous antibiotics earlier is an improvement to the medical core processes mentioned above and is able to make an impact on every parameter of the value equation and, thus, improves the value of care.

### Improvement of *status quo*

By improving on the *status quo* of a medical core process an impact on the value of care can also be achieved. Patients with acute heart failure, for example, nearly always receive so-called diuretics, which is a medication that enhances the elimination of urine and, thus, reduces water excess in the body. The earlier these medications are given, the sooner patients experience improvement of their breathing difficulties (=improvement of patient experience), the lower are mortality rates (=outcome), and the more efficient the treatment is with further care on the regular ward instead of the ED (=lower costs). Improvement of this medical core process can be achieved by educating the medical staff and providing active feedback about the time that is passing from admission to the administration of the urgently needed medication.

During discharge, patients often find it helpful to be educated about the medication they have to take. This kind of medical core process can be improved by support through in-house pharmacists who educate patients about possible side effects of their medication and exact instructions about how and when to take them. Thus, patient experience can be significantly improved.

### Focus on Quality

More than 30 years ago, Donabedian (4) explained his understanding of three different quality dimensions. Because of rising importance, one can add a fourth quality dimension, especially through the eyes of doctors and payers: indication quality or the question of the appropriateness of care. According to these quality dimensions, it is possible to review how care is organized around patients (structure quality), why treatment of a procedure is being carried out (indication quality), how well-certain practices are being executed (process quality), and what happened to the patient (outcome quality).

By focusing on these quality dimensions and constantly improving the quality of delivering care at the medical core processes, not only can performance be enhanced, but also an impact on the value of care can be achieved.

By using modern clinical pathways, variation of care delivered to patients can be reduced. Furthermore, clarity about treatment approaches and awareness of roles are raised and, thus, enhance structure quality. Reduction of variation commonly reduces costs in healthcare and, hence, positively influences the value equation.

By agreeing on certain indications for cost-intensive imaging procedures, e.g., for patients with a short self-limiting episode of fainting, these imaging procedures are being ordered when necessary and not to gain a certain level of secureness for doctors. Efficient administration of antibiotics (=process quality) in patients with severe infections in the ER has long been shown to improve survival (=outcome quality) and reduce costs due to fewer patients being treated on cost-intensive intensive care units.

**Figure 2:**
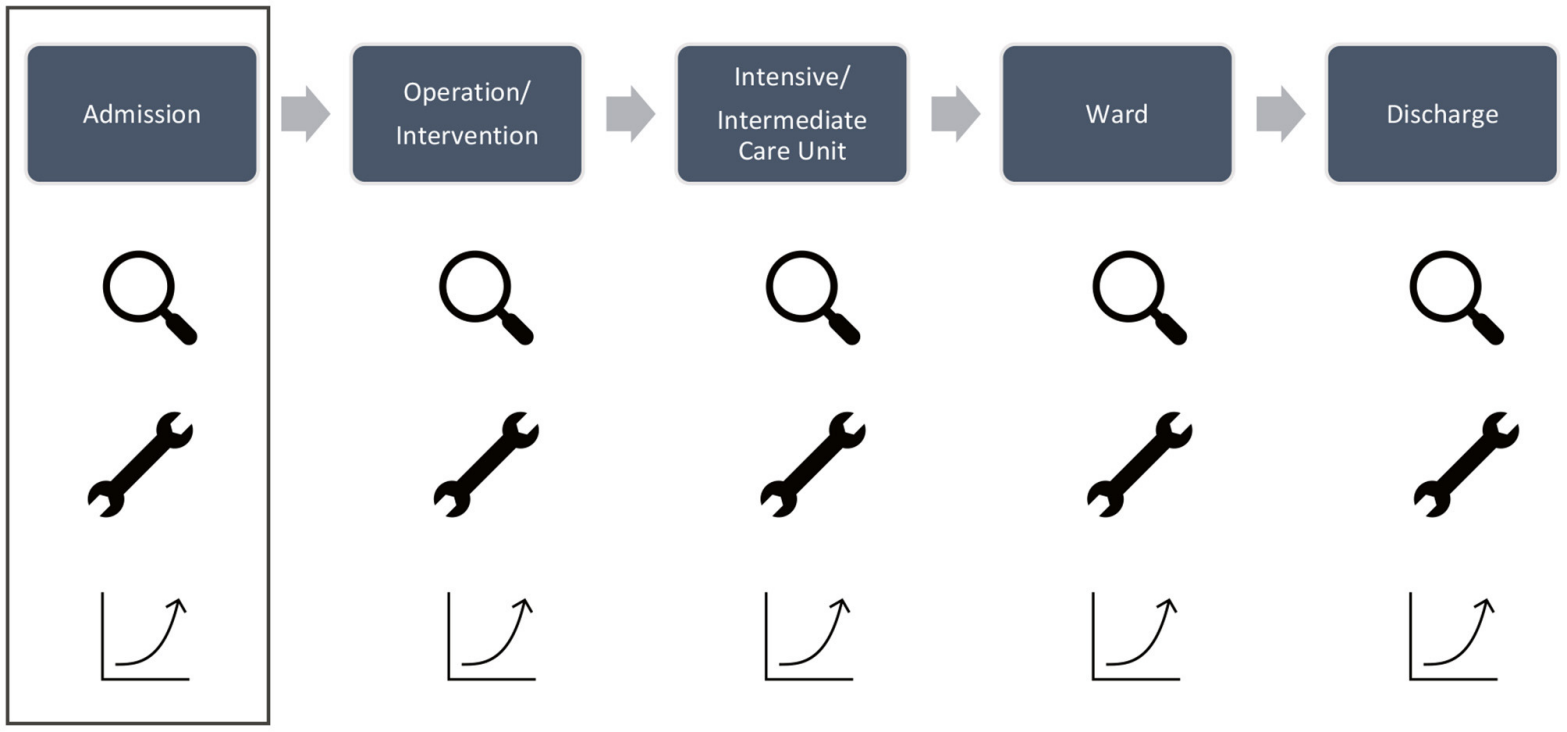
Systematic approach to improve performance and value of care at each step of the patient journey—for example, “admission” by: (1) looking for medical core processes “spyglass symbol,” e.g., the evaluation of postacute care solutions; (2) improvement of medical core processes by a set of tools (tool symbol; improvement of the *status quo*, innovation, focus on quality), e.g., the use of PACD-scoring as innovation; and (3) gaining results by permanently integrating tools in daily clinical activities (analysis symbol), e.g., reducing length of stay, therefore, costs, enhancing patient's satisfaction by providing a comprehensive evaluation of their needs and improving the value of care *via* post-acute care discharge (PACD) scoring.

By improving medical core processes along the patient journey by three different approaches, it is possible to enhance performance and generate a positive impact on the value of care. This can proceed in a generic way: first, by looking for medical core processes; second, by improving medical core processes by either innovation, improvement of the *status quo*, or focus on quality; and third, by implementation in daily practice to gain results (Figure 2).

Some of the economic results have been a reduction of length of stay of certain patient groups by 50% and more (e.g., hip and knee surgery), an increase of reimbursement/case of up to 30% (e.g., internal medicine), a reduction of lab costs of about $15,50,000 per year throughout the entire hospital, a reduction of costs of medication of about $3,00,000 per year in the Department of Internal Medicine, a reduction of costs of materials (e.g., implants and disposables) of about $10,00,000 per year throughout the entire hospital and a significant rise in patient satisfaction (e.g., Department of Neurosurgery) to above Swiss average. These economic results have been monitored closely with Key Quality Indicators (KQIs), some of which could be improved as well (e.g., one of the lowest rehospitalization rates in heart failure in Switzerland), none of which getting worse (5).

However, one should be aware of several other aspects such as the change of mindset and obstacles that need to be overcome.

## Change of Mindset: A New and Common Understanding

Besides striving for performance enhancement, looking for and improving medical core processes, and hence influencing the value of care, one should also seek a new and common understanding concerning healthcare delivery.

First, it is important to realize, communicate, and prove that raising efficiency during performance enhancement is not automatically associated with a reduction of quality. These changes the way by which employees feel about performance enhancement itself. It certainly helps clinicians and other staff in developing an open-minded attitude toward efficiency, cost, and quality in the highly sensitive and dynamic area of healthcare.

Second, performance enhancement has to go hand-in-hand with developing and monitoring KQIs, not only to realize quickly any deteriorating effect, but also to show improvement in the quality and, thus, the value of care, showing a positive impact to employees and, thereby, catalyzing future efforts by motivating medical staff.

Third, the power of data in healthcare, from its analysis to generating value, leads to a new understanding of their importance for daily business in hospitals. However, one has to be aware that a solid IT infrastructure and in-depth expertise of medical core processes to interpret these data are mandatory.

Fourth, assumptions—regardless of the hierarchical level they are made—have to be categorically challenged and replaced by proof or disproof. Thus, it can be shown and not only simply assumed that performance enhancement is able to generate a positive impact on the value of care and costs and quality in healthcare.

Finally, two often separately discussed worlds in the healthcare sector—the medical/scientific world and the management world—have to be viewed and understood as one world. Only by realizing that neither of these worlds can drive this kind of change by itself and full potential can be realized only through joint efforts.

## Obstacles, Concerns, and How They Were Overcome

Before and during the effort of hospital-wide performance enhancement, several obstacles and concerns have to be anticipated, addressed, and overcome.

The greatest concern often is compromising the quality of care on the journey to a highly efficient provider due to a lack of understanding that gaining efficiency does not consequently mean loss of quality (see above). These concerns have to be addressed by closely monitoring KQI's on the one hand and proof of even raising quality and, thus, the value of care on the other hand during the very first projects. These KQIs have to be discussed among all the key stakeholders in mandatory meetings on a regular basis.

An important obstacle often is a lack of understanding necessity and urgency to change. However, this problem can be addressed by clearly showing the dramatic changes in the healthcare landscape, rising regulations of the public authorities, and decreasing reimbursements over the years with the upcoming dilemma mentioned above. Management and medical staff have to understand each other's perspectives.

Another obstacle might be concerns of employees about the workload for the journey to come. Yet, by the focus on the reduction of length of stay as one of the very first measures, the workload for medical and administrative staff can be reduced significantly. As a consequence, assigning highly motivated employees to project teams can be carried out, so that no additional personnel has to be recruited. It would, therefore, be a mistake if management would monetize the gain of a reduced length of stay by, therefore, reducing the medical workforce. This would lead many to conclude that their apprehensions were true and potentially resist further optimizations projects.

## Discussion

The Healthcare landscape in recent days is changing fast and poses great challenges to healthcare providers, especially hospitals and their medical staff. Delivering a high quality of care with decreasing rather than increasing resources is one major challenge and the one the authors—medical practitioners with an experience of at least 15 years each in internal and emergency medicine—were striving for the last 6 years with the approach described in this article. However, by consequently striving for improved medical core processes through innovation, improvement of the *status quo*, and focusing on quality this challenge can be overcome with significant impact on the value of care, as the execution of this approach has shown (4). A change in mindset, awareness of obstacles, and a common understanding of the medical-scientific world and the management world as one world are essential.

In the healthcare environment of today, we have to be prepared for changes and different requirements, may these be the evolution of new technologies such as robotics, personalized medicine, or adverse experiences such as the global COVID-19 crisis requiring expensive reserve capacities. However, ultimately, we are serving the patients as individuals when medical help is required while being obligated to society to provide our services efficiently so care will be and stay affordable in the future.

## Data Availability Statement

The original contributions presented in the study are included in the article/supplementary material, further inquiries can be directed to the corresponding author.

## Author Contributions

All authors equally contributed to the development of the framework mentioned in the article above, the execution in daily practice, the analysis and interpretation of data, the development of this article for publication, manuscript revision, and read and approved the submitted version.

## Conflict of Interest

The authors declare that the research was conducted in the absence of any commercial or financial relationships that could be construed as a potential conflict of interest.

## Publisher's Note

All claims expressed in this article are solely those of the authors and do not necessarily represent those of their affiliated organizations, or those of the publisher, the editors and the reviewers. Any product that may be evaluated in this article, or claim that may be made by its manufacturer, is not guaranteed or endorsed by the publisher.
